# Nutritional and Anti‐Nutritional Properties of Extruded Puffed Snack Produced From Pearl Millet and Bambara Groundnut Flour Blends

**DOI:** 10.1002/fsn3.4670

**Published:** 2024-12-15

**Authors:** Oladapo Ajala, Oluwasola Abayomi Adelusi, Olatundun Esther Kajihausa, Oluseye Olusegun Onabanjo, Oyewole Olusola Bandele, Adewale Olusegun Obadina

**Affiliations:** ^1^ Department of Food Science and Technology Federal University of Agriculture Abeokuta Nigeria; ^2^ Department of Biotechnology and Food Technology, Faculty of Science University of Johannesburg, Doornfontein Campus Johannesburg South Africa; ^3^ Department of Nutrition and Dietetics Federal University of Agriculture Abeokuta Nigeria; ^4^ Department of Microbiology Babcock University Ilishan, Remo Ogun State Nigeria

**Keywords:** anti‐nutritional properties, Bambara groundnut, extruded snacks, malnutrition, nutritional attributes, pearl millet

## Abstract

This study investigates the nutritional and anti‐nutrient profiles of extrudates produced from seven formulations of pearl millet and Bambara groundnut flour in seven different ratios: 20:80, 30:70, 40:60, 50:50, 60:40, 70:30, and 80:20, with 100% pearl millet and 100% Bambara groundnut extrudates used as controls. The extrudates were processed using a twin screw extruder and analyzed for their nutritional and anti‐nutritional properties. The findings revealed a rising pattern in the content of fiber, moisture, protein, ash and fat as the substitution of Bambara groundnut increased in the extrudate. Conversely, the level of carbohydrates decreased with increased inclusion of Bambara groundnut. Our results also indicated a significant difference in the mineral composition of the pearl Millet‐Bambara groundnut extrudates, except for manganese. The values for calcium, iron, magnesium, phosphorus, sodium, copper, manganese, and potassium ranged from 38.43–67.97, 3.60–5.10, 39.64–64.65, 290.84–413.15, 10.60–21.76, 0.10–0.24, ND—0.08, and 21.51–70.45 mg/100 g, respectively. Notably, the calcium, sodium, and potassium levels in the snacks rose with higher proportions of Bambara groundnut in the extrudates. Conversely, the concentrations of iron, magnesium, phosphorus, and copper in the samples decreased as the percentage of Bambara groundnut in the formulation increased. However, the mineral levels recorded did not exceed the recommended daily intake, assuring no negative health effects upon consumption. Furthermore, the anti‐nutrient content, including trypsin inhibitor, phytate, and tannin, increased with the addition of Bambara groundnut flour, while the phenolic content decreased with increasing Bambara groundnut substitution. Overall, this study provides valuable insights for the development of nutritious snacks using locally available grains.

## Graphical abstract

The nutritional and anti‐nutrient profiles of extrudates produced from various combinations of pearl millet and Bambara groundnut was examined. The findings revealed a rising pattern in the content of fiber, moisture, protein, ash and fat as the substitution of Bambara groundnut increased in the extrudate. The anti‐nutrient content, including trypsin inhibitor, phytate, and tannin, increased with the addition of Bambara groundnut flour, while the phenolic content decreased with increasing Bambara groundnut substitution.

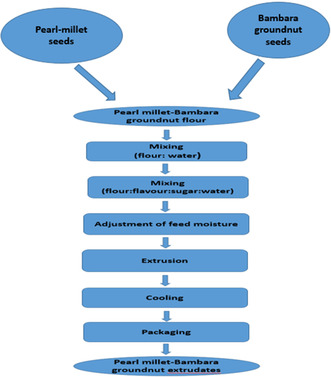

## 1 Introduction

Malnutrition is a significant issue in public health that affects individuals of all ages worldwide (Dukhi 2020). Not only does it pose a threat to public health, but it also hampers global efforts to eradicate poverty, and impede economic growth (WHO 2020). According to data provided by the World Health Organization (WHO), a substantial proportion of the world population is impacted by malnutrition (WHO 2020). Approximately, 1.9 billion adults are overweight and/or obese, with 462 million adults classified as underweight. Moreover, among children under the age of five, 41 million are overweight and/or obese, 52 million are experiencing wasting, 17 million are highly wasted, and 155 million are stunted (WHO 2020). Regrettably, malnutrition continues to pose a significant public health challenge, particularly in Africa (Nwankwo et al. 2022). About one in every seven children in sub‐Saharan Africa (SSA) experiences mortality before the age of five due to malnutrition (Walton and Allen 2011). It has been established that the eradication of malnutrition can alleviate 32% of the global disease burden (WHO 2020). These statistics underscore the pressing need for effective interventions to address these nutritional issues. Therefore, there is a need to improve nutrition, end hunger, promote healthy living, and ensure well‐being for all in the continent (Dukhi 2020). One promising approach that is gaining attention in the fight against the malnutrition epidemic in Africa is the utilization of grain, nuts, and legumes to produce several food products (Kareem et al. 2015; Adebowale et al. 2016; Jenfa et al. 2024a, 2024b). This approach aims to enhance human nutrition and promote food security across the continent.

Legumes like Bambara groundnut (*Vigna subterranean* (*L*) *verdc*) and type of millet grain like pearl millet ( *Pennisetum glaucum* ) possess significant nutritional value, making them valuable for improving human nutrition and mitigating malnutrition, especially in SSA. Pearl millet is the sixth most important type of millet grain in the world, falling just behind rice ( *Oryza sativa* ), wheat ( *Triticum aestivum* ), maize ( *Zea mays* ), barley ( *Hordeum vulgare* ), and sorghum ( *Sorghum bicolor* ). Pearl millet is a staple food for more than 90 million individuals residing in less developed nations and is substantially grown across approximately 30 million hectares of arid and semi‐arid regions in Asia and Africa (Satyavathi et al. 2021). Pearl millet is a highly nutritious grain that offers a wealth of essential nutrients, including carbohydrates, fibers, soluble and insoluble fats, ash, as well as antioxidants (Uppal et al. 2015; Adebanjo et al. 2020; Pei et al. 2022). It is also rich in minerals, including magnesium, potassium, phosphorous, manganese, zinc, iron and copper, and vitamins, such as thiamine, niacin, and riboflavin (Weckwerth et al. 2020). In terms of protein content, pearl millet is a superior source compared to other cereals like maize and rice due to its higher digestibility of proteins. This is because it has a lower content of cross‐linked prolamins (Satyavathi et al. 2021). Pearl millet has several health‐promoting benefits, including reducing anemia owing to its high iron content and managing constipation due to its high dietary fiber level (Saleem et al. 2023).

Bambara groundnut, a legume cultivated in semi‐arid savannahs and SSA, is a reliable source of food and income in the face of the negative consequences of climate change. According to Tan et al. (2020), Bambara groundnut holds significant potential in combating malnutrition globally, particularly in Africa owing to its richness in protein, amino acids, dietary fiber, and vitamins. This plant is considered a health‐beneficial food because it is rich in minerals such as potassium, zinc, magnesium, and iron, as well as amino acids, including threonine, lysine, leucine, valine, and methionine, when compared to other legumes (Tan et al. 2020; Egedigwe‐Ekeleme et al. 2023). In addition to its nutritional properties, Bambara groundnut also contains various antioxidants such as phenolic compounds, flavonoids, and tocopherols, which contribute to its antioxidant activity (Adedayo et al. 2021; Oyeyinka et al. 2021). Furthermore, Bambara groundnut exhibits antimicrobial properties, meaning it has the ability to inhibit the growth of microorganisms such as bacteria, fungi, and viruses (Ramatsetse, Ramashia, and Mashau 2023). This antimicrobial activity can help protect against food spoilage and foodborne illnesses, as well as support overall health by combating harmful pathogens in the body (Ramatsetse, Ramashia, and Mashau 2023).

The utilization of native crops like pearl millet and Bambara groundnut for producing several extruded samples holds immense promise due to their inherent nutritional richness, diversified portfolio of dietary, and resilience to challenging environmental conditions Osuna‐Gallardo et al. (2023); Bayomy et al. (2024). Despite their rich nutritional content, pearl millet and Bambara groundnut contain certain anti‐nutrients, such as trypsin inhibitors and condensed tannins, which hinder the digestion and absorption of nutrients by the plant for both animal and human consumption (Unigwe et al. 2017; Adeyeye et al. 2019). Anti‐nutritional factors encompass a group of naturally occurring compounds in foods that interfere with the absorption or utilization of nutrients, potentially posing health risks (Adeyeye et al. 2019). Understanding the impact of various flour blends on the nutritional and anti‐nutritional compositions of pearl Millet‐Bambara groundnut extrudates is of utmost importance for food scientists and consumers. Unfortunately, there is a scarcity of information pertaining to this subject matter in Africa and worldwide. Therefore, this study aimed to unravel the nutritional and anti‐nutritional compositions of extruded snacks derived from pearl millet and Bambara groundnut composite flour with the potential to provide valuable insights for enhancing the nutritional quality of food products derived from these indigenous grains.

## 2 Materials and Methods

### 2.1 Materials

**FIGURE 1:**
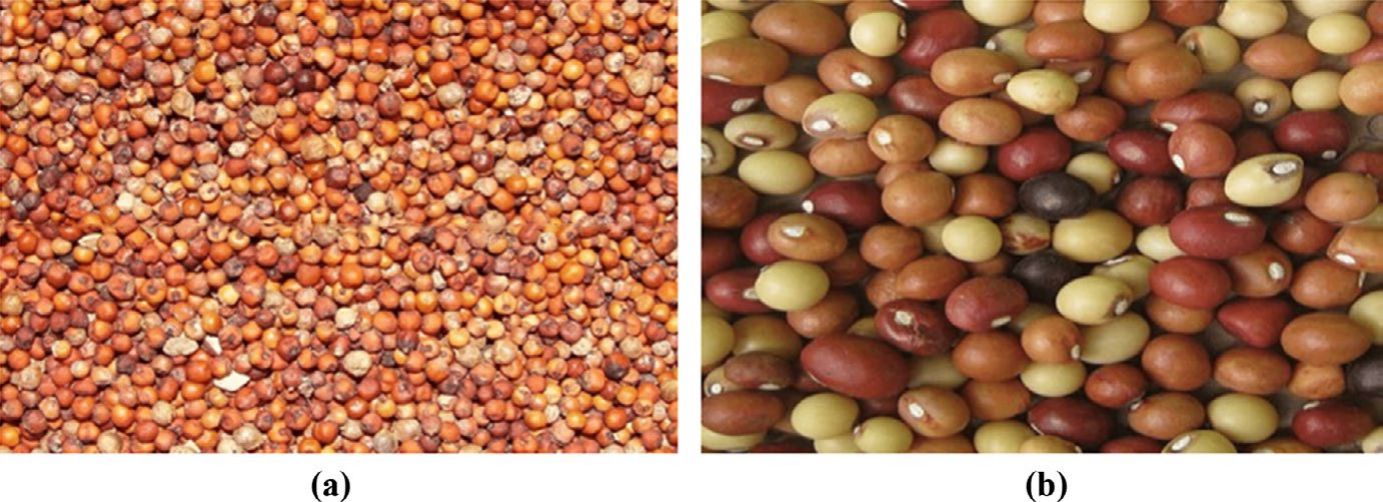
(a) Pearl‐Millet seeds, (b) Bambara groundnut seeds.

Bambara groundnut (*Vigna subterranean* (*L*) *verdc*) and pearl millet ( *Pennisetum glaucum* ) (Figure 1), and other materials such as sugar and vanilla flavor used in this present study, were procured at the Oyingbo open market in Lagos State, Nigeria. Also, the equipment used in this study, including cabinet dryer (LEEC Ltd., Serial No. 3114), fabricated dehulling machine, the fabricated dehulling machine demonstrates a dehulling efficiency of 67%–83% and a yield of 74%–80%. Each batch processes in 2 min or less, contingent on the product and debranning level. It has a Capacity (Throughput) of 120 kg/h and operates with a 5 hp. electric motor, hot air oven (model GZX9420MBE, Shanghai Boxun Experimental Co. Ltd., China), extrusion machine (XTS56‐Xtrutech, Jinan KeySong Machinery Co. Ltd., Gaoxin district, Jinan, China) with diameter, motor and average outputs of 56 mm, 130/174 kW, 1000 kJ/h, respectively, weighing scale, and attrition mill (Gu¨ven Machinery Co., Gaziantep, Turkey) were sourced from the Confectionery Processing facility located at the Federal University of Agriculture in Abeokuta (FUNAAB), Nigeria.

### 2.2 Methods

#### 2.2.1 Bambara Groundnut Flour Preparation

The procedure outlined by Chikwendu, Nwamarah, and Nnebe (2015) was applied for the manufacture of Bambara groundnut flour. Prior to initiating the process, the Bambara groundnuts were thoroughly cleaned to eliminate foreign particles. Subsequently, the cleaned Bambara groundnuts were conditioned and de‐hulled using a fabricated de‐hulling machine. The dehulled groundnuts were then separated and dried at 100°C for 20 min in a hot air oven (Shanghai Boxun Experimental Co. Ltd., China). After drying, the groundnuts were milled using a disc attrition mill (Guven Machinary Co., Gaziantep, Turkey). The resulting flour was then sifted through a 425 μm wire mesh to obtain a finer product. Finally, the flour was packaged in high‐density polyethylene bags and stored at room temperature for further analysis.

#### 2.2.2 Pearl Millet Flour Preparation

The preparation of pearl millet flour follows the procedure established by Deshpande and Poshadri (2011). In brief, millet grains were cleaned to remove any foreign material and milled into flour using a hammer mill with an 800 μm screen size (ABC Engineering, Pančevo, Serbia). The resulting flour was then sifted through a 425 μm wire mesh to obtain a finer product and stored in airtight high‐density polyethylene bags at room temperature until further analysis.

#### 2.2.3 Formulation of Pearl Millet‐Bambara Groundnut Flour Blends

Seven ratios of pearl millet and Bambara groundnut flours were combined to produce composite flour samples (Table 1). Sample PB_1_, consisting 100% of Bambara groundnut flour, and Sample PB_9_, made from 100% pearl millet flour, were used as control samples.

**TABLE 1:** Formulation of the blends pearl millet flour to Bambara groundnut flour.

S/N	Sample code	Pearl millet flour (%)	Bambara groundnut flour (%)
1	PB_1_	0	100
2	PB_2_	20	80
3	PB_3_	30	70
4	PB_4_	40	60
5	PB_5_	50	50
6	PB_6_	60	40
7	PB_7_	70	30
8	PB_8_	80	20
9	PB_9_	100	0

#### 2.2.4 Preparation of Pearl Millet‐Bambara Groundnut Extruded Puff Snacks

**FIGURE 2:**
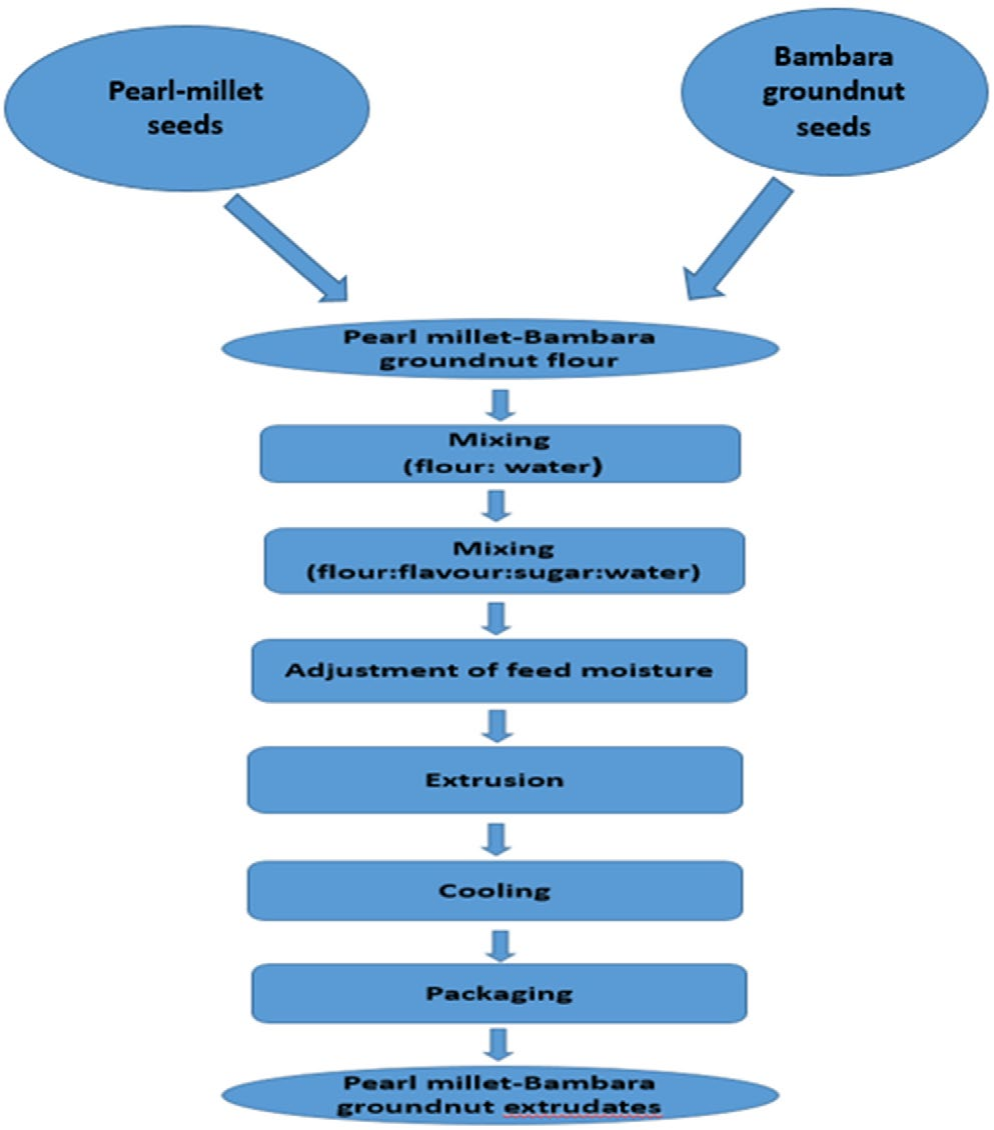
Flow chat for the production of pearl Millet‐Bambara extruded snack using modified method of Kareem et al. (2015).

The production of extruded snacks utilized a blend of pearl Millet and Bambara groundnut flour, following a modified version of the method described by Kareem et al. (2015). The process involved combining 10 kg of the composite flour with 500 g of sugar, 3 g of vanilla flavor, and 500 mL of water in the extruder's mixing chamber. The feed samples were pre‐conditioned to a moisture content of 27% dry basis, thoroughly mixed, and allowed to hydrate uniformly for 2 min in order to eliminate any areas of dryness within the raw material. The flour was extruded using an industrial twin‐screw extruder (XTS56‐Xtrutech, Jinan KeySong Machinery Co. Ltd., Gaoxin District, Jinan, China) with a diameter, motor, and average outputs of 56 mm, 130/174 kW, and 1000 kg/h, respectively. The extruder processing line was composed of four sections: mixing, transmission die, coating, and drying zones. The machine was operated at full speed under the following constant conditions: first barrel section (69°C and 50°C), second barrel section (120°C and 120°C), third barrel section (140°C and140°C), die temperature (100°C), and screw speed (900 rpm). The extruded pearl Millet‐Bambara groundnut composite flour snack was cooled to room temperature and packaged in aluminum laminate for further analysis. Figure 2 represents the flowchart for pearl millet‐Bambara groundnut extrudate production.

#### 2.2.5 Nutritional Analysis

The nutritional compositions of the pearl millet‐Bambara groundnut extrudates were determined according to the procedure outlined in AOAC (2004).

##### 2.2.5.1 Moisture Content

The method developed by AOAC (2004) was used to ascertain the moisture content of the extruded snacks.

$$\%\text{Moisture content}=\frac{M2-M1}{M3-M1}\times 100$$

where M1 − initial mass of empty dish, M2 − mass of dish + undried sample, M3 − mass of dish + dried sample.

##### 2.2.5.2 Ash Content

The ash content in the extruded snacks was determined using the oven method outlined by AOAC (2004).

$$\%\mathrm{Ash}=\frac{\left(\;\text{Weight of crucible}+\mathrm{ash}\right)-\text{weight of empty crusible}\;}{\text{Weight of sample}\;}\times 100$$

##### 2.2.5.3 Crude Fat

The method described in AOAC (2004) was employed to determine the crude fat content in the extruded snack.

$$\%\mathrm{Fat}=\frac{\left(\text{Initial weight}-\text{final weight}\right)}{\text{Weight of sample}\;}\times 100$$

##### 2.2.5.4 Crude Fiber

The methodology outlined in the AOAC (2004) was used in determining the crude fiber content in the extruded snack.

$$\%\text{Crude fiber}=\frac{M1\;}{M2}\times 100$$

where M1 − Weight loss on ignition (g) and M2 − weight of sample (g).

##### 2.2.5.5 Crude Protein

The protein content of the extruded snacks was determined using the Kjeldahl method, in accordance with the guidelines provided by AOAC (2004).

$$\%\text{Nitrogen}=\frac{\text{Titre value}\;}{\text{Weight of sample}}\times 0.01\times 0.01401\times 5\times 100$$

$$\%\text{Crude protein}=\left(\%\text{Nitrogen}\times 6.25\right)$$

##### 2.2.5.6 Carbohydrate Content

The AOAC (2004) protocol was followed to estimate the total carbohydrate content in each extruded snack blend. This method involved deducting the collective percentages of ash, crude fiber, protein, moisture, and fat from 100%.

$$\%\text{Carbohydrate}=100\%-\left(\%\mathrm{fat}+\%\text{moisture}+\%\text{crude fiber}+\%\mathrm{ash}+\%\text{protein}\;\right)$$

#### 2.2.6 Determination of Mineral Composition of Pearl Millet‐Bambara Groundnut Extruded Snack

Minerals, including Fe, Cu, Ca, Mg, K, P, Na, and Mn were analyzed following AOAC (2004) protocols. Approximately 10 g of each puffed snacks was placed in an individual crucible and heated directly to remove the organic content. Subsequently, the extrudates were transferred to a muffle furnace and exposed to a controlled temperature of 450°C for 5–7 h. The obtained ash was combined with 10 mL of diluted HCl, briefly heated, and further diluted with 100 mL of distilled water. The solution was subsequently employed for mineral analysis, as discussed below.

##### 2.2.6.1 Phosphorous Determination

The phosphorus content of the snacks was determined using the AOAC (2004) method. Dissolved in 300 mL of distilled water was 1.25 g of ammonium metavanadate and 25 g of ammonium molybdate. After warming, cooling, and diluting with 500 mL distilled water, this solution was combined with 215 mL of concentrated HCl diluted to 500 mL with water. Approximately 0.879 g of dehydrated phosphorus dihydrogen orthophosphate was dissolved in water to prepare a stock solution of phosphorus. This was achieved by adding 1 mL of concentrated HCL, diluting the solution to 200 mL with ammonium molybdate, and subsequently adding 2 mL of toluene to achieve a final concentration of 1 mg/mL for the working standard. To make various concentrations, 2 mL of the phosphorus solution was mixed with different volumes of water, including 0, 0.25, 0.5, 1, and 2 mL and the resulting solution was then transferred into a 200 mL volumetric flask. To each flask, phosphorus standard of 5 mL was added, along with molybdate mixture of 10 mL, and diluted with water of approximately 500 mL. After a 15‐min color development period, the absorbance was measured at 400 nm against a blank. A calibration curve based on absorbance and phosphorus equivalents was used to determine the extrudate's phosphorus content (mg/mL).

##### 2.2.6.2 Iron Determination

The iron content in the extruded samples was determined using the phenanthroline method, following the guidelines provided by AOAC (2004). About 0.1 g of 1,10‐phenanthroline molybdate was dissolved in 100 mL of distilled water and heated to 80°C to prepare phenanthroline solution. Furthermore, a hydroxylamine solution was prepared by dissolving 10 g of hydroxylamine in 100 mL of distilled water. Additionally, an ammonium acetate buffer solution was prepared by dissolving 250 g of ammonium acetate in 150 mL of distilled water. For the analysis, a 5 mL sample of the digested material was added to a test tube, followed by 3 mL of phenanthroline solution and 2 mL of HCl. A steam bath operating at a temperature of 600°C was employed to facilitate the boiling of the mixture for a duration of 2 min. Subsequently, an ammonium acetate buffer solution consisting of 9 mL was introduced, and the resulting mixture was further diluted to a final volume of 50 mL using distilled water. To create the calibration curve, 0, 0.25, 0.5, 1, and 2 mL of standard iron solution were pipetted into a volumetric flask (100 mL) to obtain solutions of known concentrations. The determination of the iron content of the extrudate was carried out using the resulting curve.

##### 2.2.6.3 Determination of Calcium

The titrimetric method outlined by AOAC (2004) was used to determine the calcium concentration in the extrudate. A test solution of about 10 mL was dispensed into a 250 mL conical flask, followed by the addition of potassium hydroxide (25 mL), water (25 mL), and a minimal quantity of calcium indicator. Afterwards, the mixture was titrated against a solution of an ethylenediaminetetraacetic acid (EDTA) until the endpoint was reached. The volume of EDTA used corresponds to the volume of calcium present in the solution.

##### 2.2.6.4 Determination of Potassium

The method described by AOAC (2004) was used to determine the potassium content of the extrudates. Initially, 2 mL of the ashed sample were pipetted into three separate test tubes, followed by the addition of 3 mL of water. Subsequently, 2 mL of sodium cobalt nitrite solution was added to each mixture, vigorously agitated, and left undisturbed for 45 min. After centrifugation for 15 min, the supernatant was decanted and the residue washed twice with ethanol and centrifuged again. Thereafter, distilled water (2 mL) was added to the residue and the mix was boiled for 10 min with frequent shaking to dissolve the precipitate. After cooling, 1 mL of choline hydrochloride and 2% sodium ferricyanide were added. The solution was then brought to 6 mL with distilled water, and the absorbance was measured at 620 nm against a blank.

##### 2.2.6.5 Determination of Manganese

The concentration of manganese in the snacks was determined following the AOAC (2004) method. Briefly, 2 mL of the ashed extrudate solution was transferred to three different test tubes and each tube was diluted with 3 mL of water. Subsequently, 0.5 mL concentrated H_2_SO_4_ was introduced to each test tube and heated for 1 h. Following heating, 0.1 g of sodium m‐periodate was added to each tube and heated for additional 10 min before being cooled and diluted with 10 mL of distilled water. Finally, the absorbance of each mixture was estimated at 570 nm and compared to that of the blank.

##### 2.2.6.6 Determination of Copper

The amount of copper in the puffed snacks was determined using the procedure described in AOAC (2004). In brief, 2 mL the ashed sample solution was out in three separate test tubes and 3 mL of distil water was added to the content in each test. Following this, 1 mL of versanate‐citrate solution and 0.1 mL of 1% sodium diethyldithiocarbamate was added to the mixture in the test tubes. Thereafter, the mixture was rendered alkaline by the addition of ammonia, and subsequently, 5 mL of carbon tetrachloride was added and vigorously shaken. Following this, the mixture was allowed to separate, and the absorbance of the lower layer was assessed at 440 nm with respect to a blank sample.

##### 2.2.6.7 Determination of Magnesium

The method outlined by AOAC (2004) was employed to determine the concentration of magnesium in the extruded snacks. Briefly, 2 mL of the ashed sample solution was transferred to three separate test tubes, and 3 mL of water was added to each tube. Afterwards, approximately 2 mL of a 10% sodium tungstate solution and 0.67 N H_2_SO_4_ solution were added to the contents in each test tube. The mixture in each test tube was then subjected to centrifugation for a duration of 5 min. Following this, 15 mL of the resulting supernatant was combined with 1 mL of distilled water, 1 mL of 0.05% Titan Yellow, and 1 mL of 0.1% gum ghatti. Finally, 2 mL of 10% NaOH was introduced to the mixture and the absorbance at 520 nm was determined in comparison to a blank.

##### 2.2.6.8 Determination of Sodium

The sodium content of the extruded snack was analyzed using the AOAC (2004) technique. Approximately 0.5 g of ground puffed snack sample was digested in a digester by adding a mixture of perchloric acid (HClO_4_) and nitric acid (HNO_3_) in a 1:4 ratios. Following digestion, 0.5–1.0 mL of the digested sample was extracted from the digester and diluted to a final volume of 100 mL using distilled water. The diluted sample was analyzed using an atomic absorption spectrophotometer, and the absorbance was recorded against a blank to determine the sodium concentration.

#### 2.2.7 Anti‐Nutritional Analysis of Pearl Millet‐Bambara Groundnut Extruded Snack

Anti‐nutritional, properties of the pearl Millet‐Bambara groundnut extrudates, including trypsin inhibitor activity, phytic acid, tannin, and polyphenol were determined as follows.

##### 2.2.7.1 Trypsin Inhibitor Activity

The method outlined by Kakade et al. (1974) was employed to assess the trypsin inhibitor activity in the extruded snacks. Briefly, 1 g of defatted finely ground extruded sample was extracted using 50 mL of 0.01 N NaOH solution at room temperature for 3 h. Following centrifugation at 10,000 rpm for 20 min, the resultant supernatant was utilized to determine the trypsin inhibitory activity level in the mixture. To perform the assay, aliquots ranging from 0.2 to 1 mL were pipetted into two separate test tubes and adjusted to a final volume of 2 mL using distilled water. Subsequently, 2 mL of trypsin solution was added to each tube, followed by incubation in a water bath at 39°C for 10 min. To halt the reaction, 1 mL of a 30% acetic acid solution was added to the mixture. The resulting solution's absorbance was measured at 410 nm in relation to the reagent blank. The reagent blank was prepared by adding 1 mL of acetic acid to test tubes containing trypsin and water (2 mL), followed by the addition of 5 mL of Nα‐benzoyl‐DL‐arginine‐p‐nitroanilide (BAPNA). The trypsin inhibitory activity concentration in the sample was plotted against the extract volume and extrapolated to zero. One trypsin unit was equal to a 0.01 absorbance unit increase at 410 nm per 10 mL of the reaction mixture under the specified conditions.

##### 2.2.7.2 Phytic Acid

Davies and Raid (1979) method was used to evaluate the phytate content of the extruded snacks. About 1 g of finely ground sample was extracted with 40 mL of 0.5 M HNO_3_ for 1 h and then filtered. A standard ferric chloride (FeCl_3_) solution was added to the filtrate and incubated at 100°C for 20 min, followed by another filtration. The absorbance of the standard FeCl_3_ solution and the remaining free Fe^3+^ was measured at a wavelength of 600 nm using a spectrophotometer after adding 3 mL of 0.004 M ammonium thiocyanate to the filtrate. The obtained results were converted to milligrams (mg) of phytate using the iron to phosphorus atomic ratio of 4–6, as outlined in the study conducted by Garcia‐Estepa, Guerra‐Henandez, and Garcia‐Villanova (1999).

##### 2.2.7.3 Tannin

The tannin content in puffed snacks was evaluated using the Folis‐Denis calorimetric method, following the procedure outlined by Kirk and Sawyer (1998). Initially, in 50 mL distilled water was 5 g of the extruded sample dispersed and shaken. Before being filtered through Whatman No.42 grade filter paper, the mixture was left at 28°C for 30 min. Next, the sample extract of approximately 2 mL was transferred into a volumetric flask of 50 mL, to this, the standard tannin solution of 2 mL and distilled water of 2 mL were added. Each flask volume was brought to 50 mL by adding saturated Na_2_CO_3_ solution. The mixture was then incubated for 90 min at 28°C. After incubation, the absorbance was measured at 260 nm using a spectrophotometer. A reagent blank was used to standardize the instrument at zero before taking the absorbance readings.

##### 2.2.7.4 Polyphenol

A Prussian blue assay was employed to estimate the concentration of total polyphenols in the extruded, following the methodology established by Price and Butler (1977). Initially, the ground puffed sample (0.035 g) was extracted with 5 mL of absolute methanol for 30 min. The resulting mixture was then centrifuged at 6000×*g* for 15 min, and the supernatant obtained was diluted 100 times with distilled water. The diluted supernatant was combined with 3 mL of 0.1 M FeCl_3_ in 0.1 N HCl for a duration of 3 min. Subsequently, 0.008 M potassium ferricyanide (3 mL) was added to the mixture. Following this, the absorbance of the mixture was measured at a wavelength of 720 nm using a spectrophotometer. Standard solutions of catechin hydrate were prepared to construct a standard curve with concentrations ranging from 100 to 1000 μg/L. This standard curve was then utilized to estimate the concentrations of polyphenols in the samples.

### 2.3 Validation and Accuracy

The intermediate and working standard solutions for various minerals (Fe, Cu, Ca, Mg, K, P, Na, and Mn) and antinutrients (trypsin inhibitor activity, phytic acid, tannin, and polyphenol) were created through a process of serial dilution. This involved using a 1000 mg/L standard stock solution and diluting it with deionized water. The preparation method included taking accurate measurements of both the standard solutions and reagents, as described by Ihemeje et al. (2018). To establish the calibration curve, a range of prepared working standards was analyzed for all minerals and antinutrients. Furthermore, 0.5 g samples of pearl millet and Bambara groundnut were spiked with 40% concentration of specific minerals (Fe, Cu, Ca, Mg, K, P, Na, and Mn) and anti‐nutrients (trypsin inhibitor activity, phytic acid, tannin, and polyphenol). These spiked samples were digested and analyzed under optimal conditions and the results were compared to the expected increase in the examined parameter relative to the original data Ayele, Urga, and Chandravanshi (2015).

Y=mX+c

*Y* is the dependent variable (absorbance), *X* is the independent variable (concentration), *m* is the slope of the line, and *c* is the intercept (where the line crosses the *Y*‐axis).

### 2.4 Statistical Analysis

The analyses were conducted in triplicate, and the nutritional and anti‐nutritional data obtained were analyzed using analysis of variance (ANOVA) with the Statistical Package for the Social Sciences (SPSS) version 22.0 (SPSS Inc.). Additionally, Duncan's multiple range tests was used to compare the means at a significance level of 0.05 (*p* ≤ 0.05).

## 3 Result and Discussion

### 3.1 Nutritional Composition of Pearl Millet‐Bambara Groundnut Extruded Snacks

**FIGURE 3:**
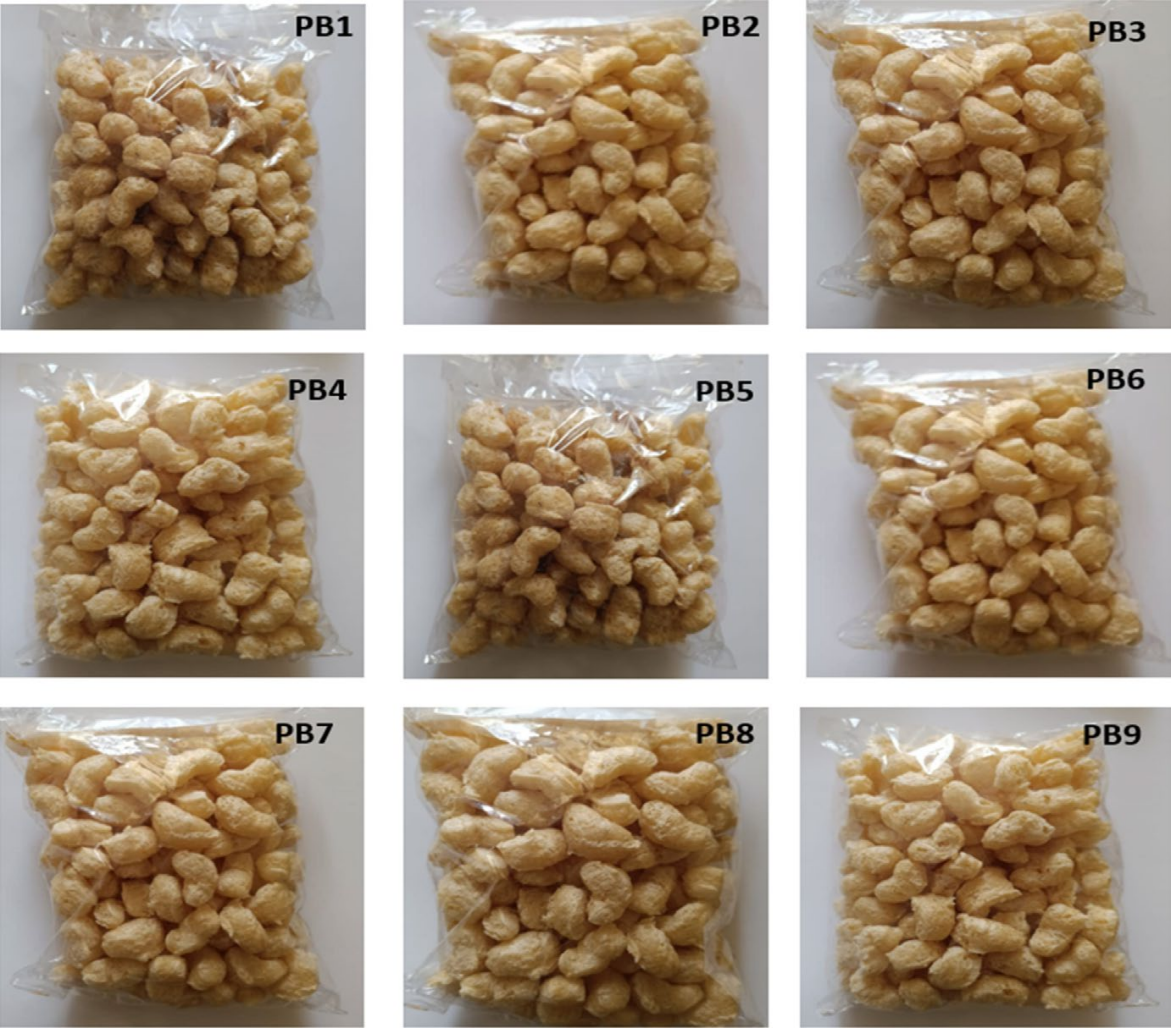
Pictures of extrudates produced from different pearl millet and Bambara groundnut flour blends. PB_1_‐100% Bambara groundnut puffed snack; PB_2_‐20% pearl millet and 80% Bambara groundnut puffed snack; PB_3_‐30% Pearl millet and 70% Bambara groundnut puffed snack; PB_4_‐40% Pearl millet and 60% Bambara groundnut puffed snack; PB_5_‐50% Pearl millet and 50% Bambara groundnut puffed snack; PB_6_‐60% Pearl millet and 40% Bambara groundnut puffed snack; PB_7_‐70% Pearl millet and 30% Bambara groundnut puffed snack; PB_8_‐80% Pearl millet and 20% Bambara groundnut puffed snack; PB_9_‐100% Pearl millet puffed snack.

**FIGURE 4:**
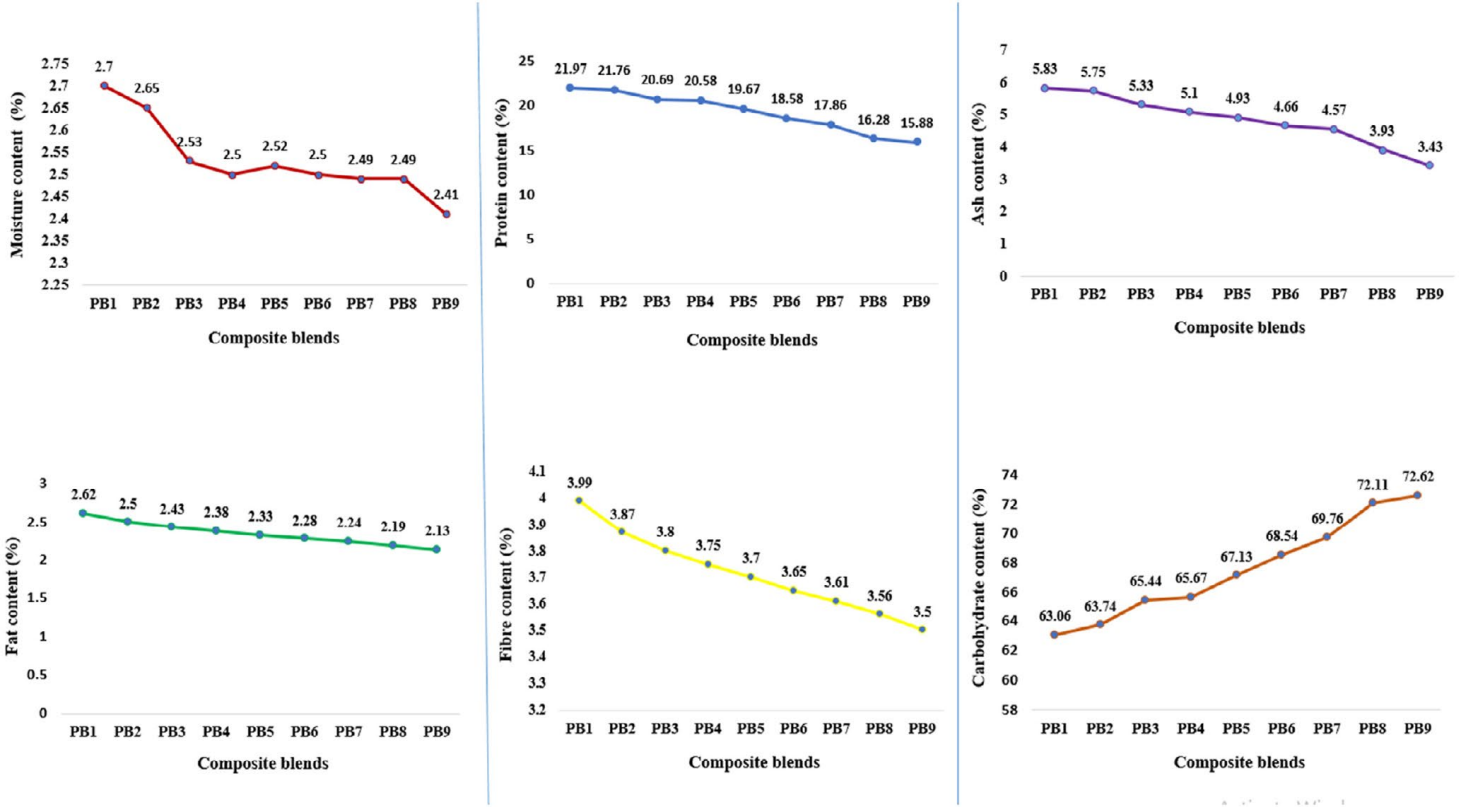
PB_1_‐100% Bambara groundnut puffed snack; PB_2_‐20% pearl millet and 80% Bambara groundnut puffed snack; PB_3_‐30% Pearl millet and 70% Bambara groundnut puffed snack; PB_4_‐40% Pearl millet and 60% Bambara groundnut puffed snack; PB_5_‐50% Pearl millet and 50% Bambara groundnut puffed snack; PB_6_‐60% Pearl millet and 40% Bambara groundnut puffed snack; PB_7_‐70% Pearl millet and 30% Bambara groundnut puffed snack; PB_8_‐80% Pearl millet and 20% Bambara groundnut puffed snack; PB_9_‐100% Pearl millet puffed snack.

The pictures of the pearl millet‐Bambara groundnut extruded puffed snacks can be found in Figure 3. The nutritional properties (carbohydrate, fiber, moisture, protein, ash and fat) of the pearl Millet‐Bambara groundnut extrudates are depicted in Figure 4. The findings revealed a rising pattern in the content of fiber, moisture, protein, ash and fat as the substitution of Bambara groundnut increased in the extrudate blends. However, the level of carbohydrates showed a decrease with the increasing inclusion of Bambara groundnut. The moisture content of the extruded samples ranged from 2.41% to 2.7%. The control sample, PB_1_, and sample PB_2_ displayed the highest moisture content, measuring 2.7% and 2.65%, respectively, while samples PB_7_, PB_8_, and PB_9_ exhibited the lowest moisture contents, with values of 2.49%, 2.49%, and 2.41% respectively. The moisture levels recorded in this current study were lower than the values reported by Adebanjo et al. (2020) for extruded samples made from flour blends of carrot ( *Daucus carota* ) and pearl millet ( *Pennisetum glaucum* ), which were in the range of 8.0%–8.16%. This discrepancy is not surprising, considering that carrot is a root vegetable with a high water content compared to Bambara groundnut (Boadi et al. 2021). Sobukola, Babajide, and Ogunsade (2012) confirmed that products that possess a higher moisture content have a shorter lifespan in comparison to those with lower moisture content, indicating the favorable shelf lives of extrudates made from pearl millet and Bambara groundnut.

The protein content of the extrudates increases proportionally with the amount of Bambara groundnut incorporated into their formulation. Among the analyzed samples, PB_1_ and PB_2_ demonstrated the highest protein content of 21.97% and 21.76%, while samples PB_8_ and PB_9_, exhibited the lowest protein concentrations at 16.28% and 15.88%, respectively. Previous studies conducted by and Kocherla, Aparna, and Lakshmi (2012) and Adebowale et al. (2016) have also shown that increasing the proportion of protein‐rich ingredients in extruded snack formulations results in a higher protein content of the produced food. This finding is consistent with the observation that Bambara groundnut flour, when included, increases the protein content of the snacks. The protein content of leguminous plant seeds like Bambara groundnut is well documented (Adegbanke, Duyilemi, and Oluwajuyitan 2019; Tan et al. 2020). The results obtained from the protein content analysis further support the notion that adding various amounts of Bambara groundnut into extrudates can significantly enhance their protein content. Consequently, this has the potential to effectively address protein‐ energy malnutrition, especially in developing countries where access to animal proteins is limited due to their high cost.

The fat content of the extrudates demonstrated an increasing pattern as the substitution of Bambara groundnut in the blends increased. Among the samples, PB_1_ and PB_2_ exhibited the highest fat contents, measuring 2.62% and 2.52%, respectively. Conversely, PB_8_ and PB_9_ displayed the lowest fat contents, measuring 2.19% and 2.13%, respectively. A lower fat content was observed in this study compared to a report by Honi, Mukisa, and Mongi (2017) on orange‐fleshed sweet potatoes (OFSP) and Bambara groundnut extruded snacks, which had a fat content ranging from 4.2% to 12.74%. Severalstudies have reported that pearl millet has a moderately low fat content (Obadina et al. 2017; Krishnan and Meera 2018). Olaleye, Adeyeye, and Adesina (2013) and Sade (2009) further confirmed that the fat content (6.99%) of dehulled Bambara groundnut is significantly higher than that of pearl millet grain (5.7%). It is worth noting that a high fat content in extrudates may be unfavorable for storage stability due to the increased tendency for rancidity. The ash content of the extrudates ranged from 3.43% to 5.83% and increased with increasing Bambara groundnut inclusion. However, the ash contents of the extrudate samples were not significantly different.

It was observed that the fiber content of the extrudates increased as the amount of Bambara groundnut substitute increased, ranging from 3.50% to 3.99%. The reported fiber levels in this study are consistent with the values (0.32%–4.78%) recorded by Ogunmuyiwa et al. (2017) in extruded snacks produced from flour blends of Bambara groundnut, cassava, and corn bran. Interestingly, the authors attributed the higher levels of crude fiber in the snacks to the high proportions of Bambara groundnut flour and corn bran. A fiber‐rich diet is essential for nutritional reasons because it helps to improve glucose tolerance and digestive health, reduce blood sugar levels, and lower cholesterol levels, as reported by Bosede and Bamidele (20232023). The carbohydrate content in samples PB_8_ and PB_9_ was the highest, at 72.11% and 72.672%, respectively, whereas PB_1_ and PB_2_ exhibited the lowest carbohydrate levels, at 63.06% and 63.74%, respectively. Remarkably, the substitution of pearl millet led to an increase in carbohydrate content in the extrudates, indicating that it is an excellent source of energy. This finding is supported by Akeem et al. (2023), who recorded carbohydrate levels of 67.60%–72.96% in gluten‐free crackers made from composite flour of germinated pearl millet ( *Pennisetum glaucum* ), defatted tigernut ( *Cyperus esculentus* ), and defatted sesame seed ( *Sesamum indicum* ). It also corroborates with Jackson and Mugula's (2022) study on the nutritional composition of soybean‐pearl millet‐cowpeas instant porridge flour extrudates, wherein the carbohydrate content varied between 69.4 and 83.3 g/100 g. The authors further revealed that the carbohydrate content of the extruded snacks declined as the amount of pearl millet in the formulations was reduced.

### 3.2 Calibration Curve for Minerals and Anti‐Nutrients

Table S1 presents the wavelengths, correlation coefficient (*R* ^2^), and calibration curve equations used for mineral analysis in the extrudates, while Table S2, shows the wavelengths, correlation coefficient (*R* ^2^), and calibration curve equations for trypsin inhibitor activity, phytic acid, tannin and polyphenol. The wavelengths for P, Fe, K, Mn, Cu, Mg, and Na measurements were 400, 248, 600, 570, 440, 520, and 440 nm, respectively (Table S1). In addition, the wavelengths for the anti‐nutrient factors, including trypsin inhibitor, phytic acid, tannin, and polyphenol were 410, 600, 600, and 720 nm, respectively (Table S2). The resulting correlation coefficients ranged from 0.9598 to 0.9996 for minerals (Table S1) and 0.9722 to 0.9985 for anti‐nutrients (Table S2), indicating excellent linearity in the calibration curves (Figures S1 and S2). The strong relationship between absorbance and mineral concentration is evident from the correlation coefficients (*R* ^2^) of the calibration curves for all minerals. Likewise, the calibration curves for the anti‐nutrients demonstrate a robust correlation between absorbance and the two anti‐nutrients, as indicated by their correlation coefficients (*R* ^2^).

### 3.3 Mineral Composition of Pearl Millet‐Bambara Groundnut Extruded Snack

Table 2 displays the mineral compositions of the puffed samples. The results indicate a significant (*p* < 0.05) difference in all mineral compositions of the pearl Millet‐Bambara groundnut extrudates, except for Manganese (which was only detected in samples PB_8_ and PB_9_). Calcium, iron, magnesium, phosphorus, sodium, copper, manganese, and potassium values ranged from 38.43–67.97, 3.60–5.10, 39.64–64.65, 290.84–413.15, 10.60–21.76, 0.10–0.24, ND–0.08, and 21.51–70.45 mg/100 g, respectively. The calcium, sodium, and potassium content of the extruded snack increased as the Bambara groundnut substitution increased, with the highest levels recorded in extruded samples PB_1_ and PB_2_, while the lowest levels were found in samples PB_8_ and PB_9_. The composition of Bambara groundnut seeds, which includes high levels of calcium, sodium, and potassium, is responsible for the observed pattern, as reported by Oyeleke, Afolabi, and Isola (2012). The calcium values (38.43–67.97 mg/100 g) observed in this study were found to be significantly higher compared to the values (1.49–2.60 mg/100 g) reported by Oluwamukomi, Oluwajuyitan, and Makinde (2021) for Ipeke agbado, traditional maize‐based snacks, fortified with Bambara groundnut flour. In contrast to the findings of the present study, the authors of the aforementioned study also observed elevated levels of sodium (136–183.5 mg/100 g) and potassium (194.50–204.50 mg/100 g) in the snacks, which demonstrated a decrease in percentage as the inclusion of Bambara groundnut in the snacks increased. The variation in the mineral levels could be attributed to the snack's flour composition and the extrusion process. Calcium is an essential mineral vital for the proper functioning of various physiological processes in the body such as cardiac muscle function, blood coagulation, and regulation of cell permeability (Lopez‐Martinez et al. 2009). Sodium is a crucial element for regulating pH, maintaining the osmotic balance of body fluids, controlling muscle and nerve excitability, and promoting normal glucose absorption and protein retention during growth.

**TABLE 2:** composition (Mg/100 g) of pearl Millet‐Bambara groundnut extruded snack.

Sample code	Calcium	Iron	Magnesium	Phosphorus	Sodium	Copper	Manganese	Potassium
PB_1_	67.97 ± 0.06^a^	3.60 ± 0.10^e^	39.64 ± 0.11^i^	290.84 ± 0.62^i^	21.76 ± 0.54^a^	0.10 ± 0.01^e^	ND	70.45 ± 0.19^a^
PB_2_	63.78 ± 0.03^b^	4.76 ± 0.05^d^	43.76 ± 0.31^h^	301.20 ± 0.54^h^	20.01 ± 0.08^b^	0.12 ± 0.01^d^	ND	65.76 ± 0.16^b^
PB_3_	59.54 ± 0.03^c^	4.82 ± 0.01^cd^	47.24 ± 0.23^g^	340.69 ± 0.19^g^	19.16 ± 0.33^c^	0.15 ± 0.01^c^	ND	54.40 ± 0.04^c^
PB_4_	57.40 ± 0.06^e^	4.86 ± 0.04^bcd^	52.28 ± 0.27^f^	360.82 ± 0.11^f^	18.12 ± 0.19^d^	0.16 ± 0.03^bc^	ND	47.50 ± 0.32^d^
PB_5_	57.84 ± 0.04^d^	4.92 ± 0.06^bc^	66.83 ± 0.02^a^	417.18 ± 0.44^a^	17.25 ± 0.21^e^	0.18 ± 0.01^b^	ND	40.61 ± 0.23^e^
PB_6_	44.68 ± 0.58^g^	4.93 ± 0.07^bc^	57.49 ± 0.30^e^	400.95 ± 0.51^e^	16.76 ± 0.39^e^	0.22 ± 0.02^a^	ND	37.72 ± 0.14^f^
PB_7_	44.94 ± 0.03^f^	4.94 ± 0.02^b^	58.36 ± 0.20^d^	405.93 ± 0.25^d^	14.34 ± 0.21^f^	0.23 ± 0.00^a^	ND	34.35 ± 0.16^g^
PB_8_	43.71 ± 0.04^h^	5.06 ± 0.02^a^	60.70 ± 0.17^c^	409.74 ± 0.70^c^	12.42 ± 0.06^g^	0.22 ± 0.01^a^	0.05 ± 0.01^a^	29.88 ± 0.05^h^
PB_9_	38.43 ± 0.11^i^	5.10 ± 0.08^a^	64.65 ± 0.12^b^	413.15 ± 0.51^b^	10.60 ± 0.25^h^	0.24 ± 0.01^a^	0.08 ± 0.03^a^	21.51 ± 0.34^i^

*Note:* Mean values with different superscripts within the same column are significantly different (*p* < 0.05). PB_1_‐100% Bambara groundnut puffed snack; PB_2_‐20% pearl millet and 80% Bambara groundnut puffed snack; PB_3_‐30% Pearl millet and 70% Bambara groundnut puffed snack; PB_4_‐40% Pearl millet and 60% Bambara groundnut puffed snack; PB_5_‐50% Pearl millet and 50% Bambara groundnut puffed snack; PB_6_‐60% Pearl millet and 40% Bambara groundnut puffed snack; PB_7_‐70% Pearl millet and 30% Bambara groundnut puffed snack; PB_8_‐80% Pearl millet and 20% Bambara groundnut puffed snack; PB_9_‐100% Pearl millet puffed snack.

The potassium content, ranging from 21.51 to 70.45 mg/100 g, falls significantly short of the reference nutrient intake (RNI) for potassium. According to COMA (1991), the recommended daily intake of *K* for adults aged 18 and above, regardless of gender, is 3500 mg.

In contrast to the observed trend in calcium, sodium, and potassium levels in the snacks, the concentrations of iron, magnesium, phosphorus, and copper in the samples decreased as the percentage of Bambara groundnut in their formulation increased. The highest levels of these elements were found in puffed samples PB_9_ and PB_8_, while the lowest concentration was recorded in samples PB_1_ and PB_2_. Among the evaluated minerals, copper exhibited low values ranging from 0.10 to 0.24 mg/100 g. The levels of iron, magnesium, phosphorus, and copper observed in this current study are significantly higher compared to the values of 0.21–0.30, 0.82–1.21, 0.26–0.33, 0.10–0.15 mg/100 g reported by Oluwamukomi, Oluwajuyitan, and Makinde (2021) in their investigation of Ipeke agbado (Nigerian traditional maize‐based snacks) fortified with Bambara groundnuts flour. The magnesium content (39.64–64.65 mg/100 g) reported in this study falls within the range found in all extrudate samples. This can be compared to findings from COMA, which established a RNI of 300 mg/day for adult males and 270 mg/day for adult females (COMA 1991). For infants and children, the RNI varies from 55 to 280 mg/day. The observed phosphorous content (290.84–413.15 mg/100 g) aligns closely with the recommendation made by COMA, which established a RNI of 550 mg/day for phosphorus in adults aged 19–50 years, regardless of gender.

Similar to our study, the amount of copper in the Ipekere agbado decreased slightly as the percentage of Bambara groundnut increased in the extrudates composite flour, while the distribution of iron, magnesium, and phosphorus in the snacks did not follow a consistent pattern.

In the present study, it was observed that the iron content in the extrudates exhibited an increase with the progressive incorporation of pearl millet into the blend, ranging from 3.60 to 5.10 mg/100 g. Samples PB8 and PB9 exhibited the maximum iron concentrations (5.06 and 5.10 mg/100 g, respectively), while samples PB1 and PB2 showed the minimum levels (3.60 and 4.72 mg/100 g, respectively). These values are lower than the recommended daily iron intake, which is 8.7 mg for males aged 11–18 years and 6.7 mg for males aged 19 years and older (COMA 1991). According to a study conducted by Jackson and Mugula (2022) on the nutritional quality of instant porridge flour made from extruded soybean‐pearl millet‐cowpeas, it was found that the iron content in the porridge flours varied from 8.4 to 14 mg/100 g, surpassing the findings of the current study. The authors further confirmed that the levels of iron in the extrudates declined as the proportion of pearl millet reduced and legumes (cowpeas and soybeans) were integrated into all formulations. The high iron level detected in samples PB_8_ and PB_9_ might be due to the fact that pearl millet contains more iron than legumes, as explained by Jackson and Mugula (2022). Interestingly, only samples PB_8_ (80% pearl millet and 20% Bambara groundnut) and PB_9_ (100% pearl millet) contained manganese with values varying from 0.05 to 0.08 mg/100 g. This result corroborates the study of Olaleye, Adeyeye, and Adesina (2013), who evaluated the seed parts of Bambara groundnut and found no detection of manganese in any of the samples. This suggests that the presence of pearl millet contributed to the manganese content of the extrudates.

### 3.4 Anti‐Nutritional Composition of the Pearl Millet‐Bambara Groundnut Extruded Snacks

**FIGURE 5:**
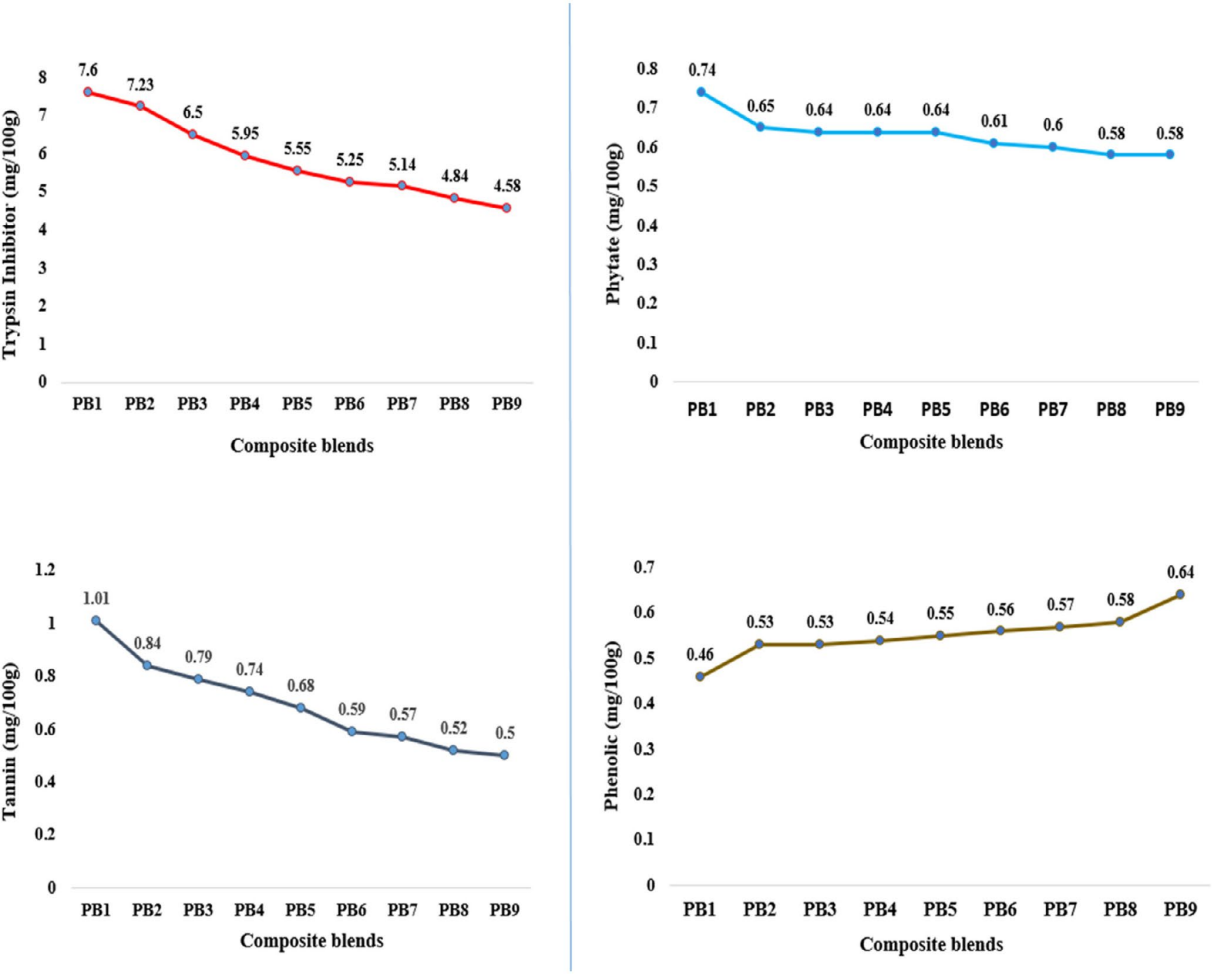
PB_1_‐100% Bambara groundnut puffed snack; PB_2_‐20% pearl millet and 80% Bambara groundnut puffed snack; PB_3_‐30% Pearl millet and 70% Bambara groundnut puffed snack; PB_4_‐40% Pearl millet and 60% Bambara groundnut puffed snack; PB_5_‐50% Pearl millet and 50% Bambara groundnut puffed snack; PB_6_‐60% Pearl millet and 40% Bambara groundnut puffed snack; PB_7_‐70% Pearl millet and 30% Bambara groundnut puffed snack; PB_8_‐80% Pearl millet and 20% Bambara groundnut puffed snack; PB_9_‐100% Pearl millet puffed snack.

Figure 5 presents data on the anti‐nutritional properties of the extruded snacks. The levels of trypsin inhibitor (4.58–7.60 mg/100 g), phytate (0.58–0.74 mg/100 g), and tannin (0.50–1.01 mg/100 g) in the snacks increased as the proportion of Bambara groundnut in the composite flour increased. Among the samples, PB_1_ and PB_2_ exhibited the highest levels of these anti‐nutritional compounds, while samples PB_8_ and PB_9_ had the lowest. Significant differences were observed in the trypsin inhibitor and tannin contents among the snacks, but not in the phytate content. The phytate and tannin levels observed in this study are lower than those reported by Eze et al. (2020), who found that snacks made from a blend of charamenya and sorghum flour had phytate and tannin contents ranging from 22 to 67 and 1 to 87 mg/100 g, respectively. However, this finding contradicts the findings of Tajoddin, Shinde, and Lalitha (2011), who affirmed that legumes, including Bambara groundnut, have a high phytate content that reduces the bioavailability of essential minerals and proteins by forming insoluble complexes. Our results for trypsin inhibitor are lower than the values (7.11–17.94 mg/100 g) reported by Adeleke, Adiamo, and Fawale (2017) for raw Bambara groundnut seeds, which may explain the higher levels of trypsin inhibitor in samples with higher Bambara groundnut contents. Conversely, the phenolic content in the extruded samples increased as the percentage of Bambara groundnut decreased. The values ranged from 0.46 to 0.64 mg/100 g, with samples PB_1_ (0.46 mg/100 g) and PB_2_ (0.53 mg/100 g) exhibiting the lowest phenolic content, while samples PB_8_ (0.58 mg/100 g) and PB_9_ (0.64 mg/100 g) had the highest values.

The various anti‐nutritional properties observed in this study may be attributed to the processing (extrusion) conditions during the preparation of the extrudates. Previous research has shown that extrusion processing enhances the bioavailability of minerals by deactivating anti‐nutrients (Sharif, Rizvi, and Paraman 2014; Paraman, Wagner, and Rizvi 2012; Duguma et al. 2021). The variation in the anti‐nutritional compositions observed among the extrudates may also be linked to differences in their flour composition, as revealed by Simwaka et al. (2017). Anti‐nutrients such as phytic acid and tannic acid can form insoluble complexes with minerals such as zinc, iron, magnesium, and calcium, potentially leading to mineral deficiencies (Nikmaram et al. 2017). Additionally, anti‐nutrients like trypsin inhibitors can impede digestive enzyme activity. Studies conducted by Abiodun and Adepeju (2011) have demonstrated that boiling and dehulling processes can significantly reduce the levels of anti‐nutrients in Bambara groundnut flour, while Olaleye, Adeyeye, and Adesina (2013) have recommended dehulling Bambara groundnut, particularly for infant food supplements, as it concentrates nutrients while reducing anti‐nutrients. Tannins not only decrease palatability but can also harm the intestinal tract and promote carcinogenesis (Makkar and Becker 1998). The noticeable decrease in tannin levels in the extrudates indicates an improvement in protein bioavailability.

## 4 Conclusion

This study investigated the nutritional and anti‐nutritional characteristics of puffed snacks made from a combination of pearl millet and Bambara groundnut composite flour. Among the different extrudates, puffed snacks PB_1_ (100% Bambara groundnut) and PB_2_ (80% Bambara groundnut and 20% pearl millet) exhibited superior nutritional profiles, with higher protein, fiber, and ash content. Additionally, sample PB_8_ exhibited increased concentrations of key nutritional composition and minerals, including iron, magnesium, phosphorus, copper, and manganese. However, the mineral levels recorded did not exceed the recommended daily intake, assuring no negative health effects upon consumption. Also, sample PB8 demonstrated enhanced anti‐nutritional components, such as trypsin inhibitor, phytate, and tannin, which contribute to addressing malnutrition. Thus, for enhancing dietary quality and promoting overall health and well‐being in African populations and worldwide, it is recommended to increase the proportion of Bambara groundnut in the pearl Millet‐Bambara groundnut extrudates, with samples PB_1_ and PB_2_ as promising choices owing to their elevated levels of protein, ash, and fiber, as well as essential minerals like calcium, potassium, and sodium. In conclusion, the utilization of pearl millet and Bambara groundnut extrudate products holds significant promise for combating malnutrition and promoting sustainable nutrition in developing countries. Finally, research and initiatives aimed at optimizing the production, distribution, and acceptance of these extrudate products are needed to understand their full potential.

## Author Contributions

**Oladapo Ajala:** conceptualization, methodology, data curation, formal analysis, writing – original draft. **Oluwasola Abayomi Adelusi:** investigation, visualization, formal analysis, writing – review and editing. **Olatundun Esther Kajihausa:** methodology, conceptualisation, supervision. **Oluseye Olusegun Onabanjo:** investigation, project administration, visualization. **Oyewole Olusola Bandele:** methodology, data curation, investigation. **Adewale Olusegun Obadina:** investigation, funding acquisition, supervision, project administration, writing – review and editing.

## Ethics Statement

The authors have nothing to report.

## Conflicts of Interest

The authors declare no conflicts of interest.

## Supporting information


Data S1.

## Data Availability

Data will be made available upon request from the corresponding author.
