# Supplementary material for: Nutritional and Anti‐Nutritional Properties of Extruded Puffed Snack Produced From Pearl Millet and Bambara Groundnut Flour Blends
Source: Food Sci Nutr. 2024 Dec 15;13(1):e4670. doi: 10.1002/fsn3.4670 (PMC11716987; doi:10.1002/fsn3.4670)
Supplement: Supplementary file 1 — Data S1. [file FSN3-13-e4670-s001.docx]

**Supplementary materials**

Table S1: Analytical conditions and calibration curves for Fe, Cu, Ca, Mg, K, P, Na, and Mn analysis by [Atomic Absorption Spectrometry](https://www.sciencedirect.com/topics/food-science/atomic-absorption-spectroscopy).

| Mineral element | Wavelength (nm) | Correlation coefficient  (R^2^) | Calibration curve equation |
| --- | --- | --- | --- |
| Phosphorous | 400 | 0.993 | y = 0.0197x - 0.0008 |
| Iron | 248 | 0.9947 | y = 0.0537x - 0.0128 |
| potassium | 600 | 0.9993 | y = 0.0546x-0.0121 |
| manganese | 570 | 0.9996 | y = 0.4922x - 0.1342 |
| copper | 440 | 0.9598 | y = 0.1461x + 0.1121 |
| magnesium | 520 | 0.9802 | y = 0.047x - 0.0229 |
| sodium | 440 | 0.9828 | y = 0.0623x + 0.1747 |

Table S2: Analytical conditions and calibration curves for trypsin inhibitor activity, phytic acid, tannin and polyphenol analysis by [Atomic Absorption Spectrometry](https://www.sciencedirect.com/topics/food-science/atomic-absorption-spectroscopy).

| Anti-nutrient | Wavelength (nm) | Correlation coefficient  (R^2^) | Calibration curve equation |
| --- | --- | --- | --- |
| Trypsin inhibitor | 410 | 0.9722 | y = 0.037x + 0.3414 |
| Phytic acid | 600 | 0.9792 | y = 0.122x + 0.0536 |
| Tannin | 600 | 0.9985 | y = 0.0728x + 0.0938 |
| Polyphenol | 720 | 0.9778 | y = 0.1201x - 0.0007 |

Figure S1: Calibration curves for determination of Fe, Cu, Ca, Mg, K, P, Na, and Mn.

Figure S2: Calibration curves for determination of for trypsin inhibitor activity, phytic acid, tannin and

Polyphenol.
